# Laparoscopic-Assisted Removal of Two Video Capsule Endoscopy Cameras Retained for More Than Two Years

**DOI:** 10.7759/cureus.64816

**Published:** 2024-07-18

**Authors:** Miranda Daye, Paige E Bonner, Moustapha Doulaye, Parth Patel

**Affiliations:** 1 Dr. Kiran C. Patel College of Osteopathic Medicine, Nova Southeastern University, Tampa, USA; 2 Surgery, Tampa General Hospital Crystal River, Crystal River, USA

**Keywords:** laparoscopic surgery, video capsule enteroscopy, small bowel stricture, subacute small bowel obstruction, capsule retention, video capsule endoscopy (vce)

## Abstract

Video capsule endoscopy (VCE) is used to evaluate the gastrointestinal tract, particularly the small bowel for obscure bleeding, Crohn’s disease, and tumors. A rare complication of VCE is the retention of the pill camera. With the expanding use of VCE, it’s important to consider the pathology that may lead to retention and approach to treatment. VCE for subacute or intermittent bowel obstruction is considered a contraindication due to the increased risk of retention, however, it may also identify significant pathology. Capsule retention should be treated promptly to prevent complications such as acute small bowel obstruction (SBO) and perforation. This case describes a 51-year-old female who presented with retention of two VCE cameras in the bowel for multiple years. She had intermittent abdominal pain and partial SBOs before the retention. She underwent a successful laparoscopic-assisted surgery removing the two endoscopy cameras and resection of the stenosed small bowel. This case sheds light on the challenges and opportunities in the management of VCE and capsule retention.

## Introduction

A key diagnostic tool in screening and diagnosing gastrointestinal (GI) pathology is endoscopy and colonoscopy. Traditional endoscopies require a fiber optic camera that is inserted into the mouth or anus to visualize the GI tract [1]. Video capsule endoscopy (VCE) is a noninvasive diagnostic tool in which the patient ingests a pill-sized camera that provides photo and video footage as it passes through the GI tract. It was first approved by the US Food and Drug Administration (FDA) in 2001. Up to 60,000 images were captured, and it took 30-90 minutes to review [2]. It is most often used to evaluate small bowel pathologies, including obscure GI bleeding, Crohn’s disease, and polyposis syndromes. Though it does not provide therapeutic benefit, it allows visualization between the lengths that upper endoscopy and colonoscopy are unable to reach. A rare complication, occurring in about 1.4% of patients, is capsule retention [3]. Some causes of capsule retention are known or suspected inflammatory bowel disease (IBD), subacute small bowel obstruction (SBO), and tumors [4]. Techniques for removing capsules include endoscopy and surgery depending on the location of the capsule. Though device-assisted endoscopy has a high success rate, surgery is often the definitive treatment for the underlying pathology [5]. Here, we present a case of laparoscopic-assisted removal of two capsules retained for two and a half years in a patient without evidence of IBD.

## Case presentation

**Figure 1:**
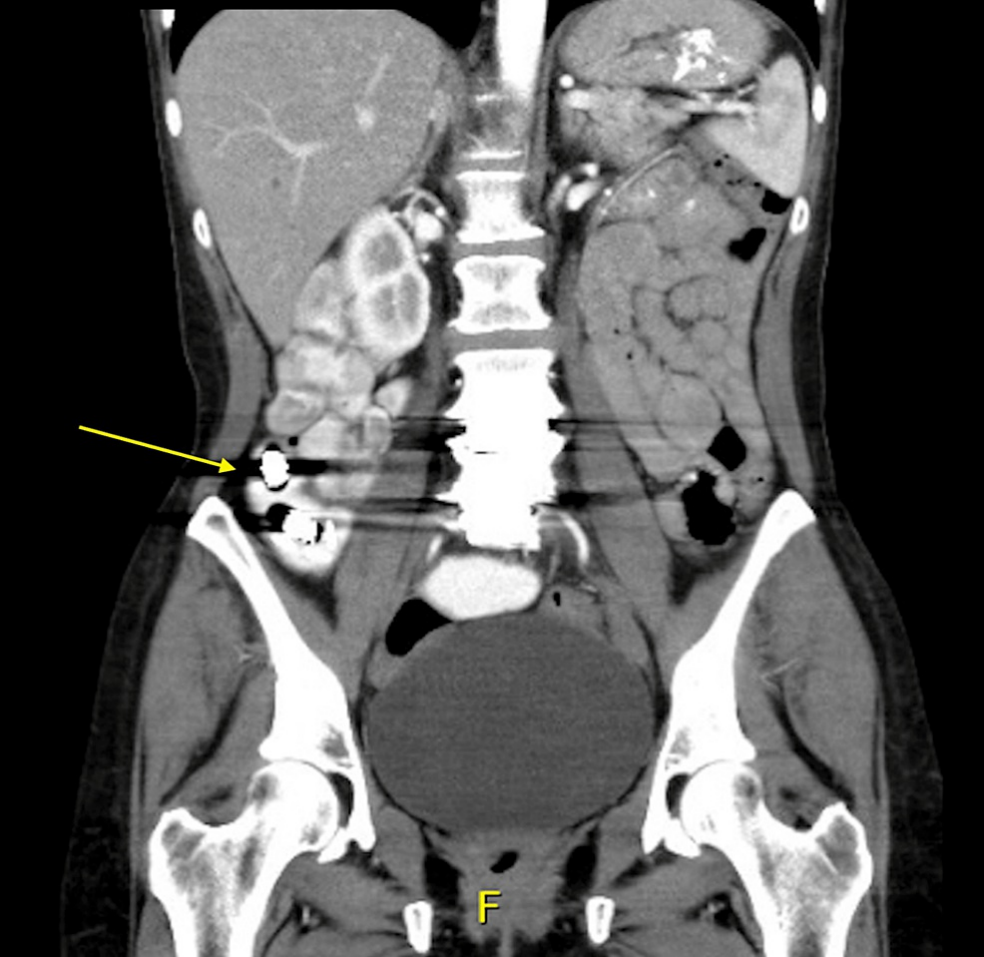
CT coronal view with the arrow pointing to two video capsules in the distal small bowel.

A 51-year-old female presented to the surgery clinic following an emergency room visit for intermittent abdominal pain and a CT abdomen showing two metallic foreign bodies in the distal small bowel (Figure 1). The patient had two VCE studies two years before this visit for her abdominal symptoms. Before the first VCE, she had a patency capsule that passed successfully. Her medical history included intermittent abdominal pain for approximately five years. The pain was worse with eating and associated with nausea, vomiting, and poor appetite. Over these five years, she was admitted to the hospital several times for intermittent SBOs. One admission was treated with exploratory laparotomy, with findings compatible with a partial small bowel volvulus but otherwise unremarkable. Her surgical history entails anterior lumbar interbody fusion. She has a past medical history of generalized anxiety disorder, major depressive disorder, tobacco dependence, and gastroesophageal reflux disorder. 

**Figure 2:**
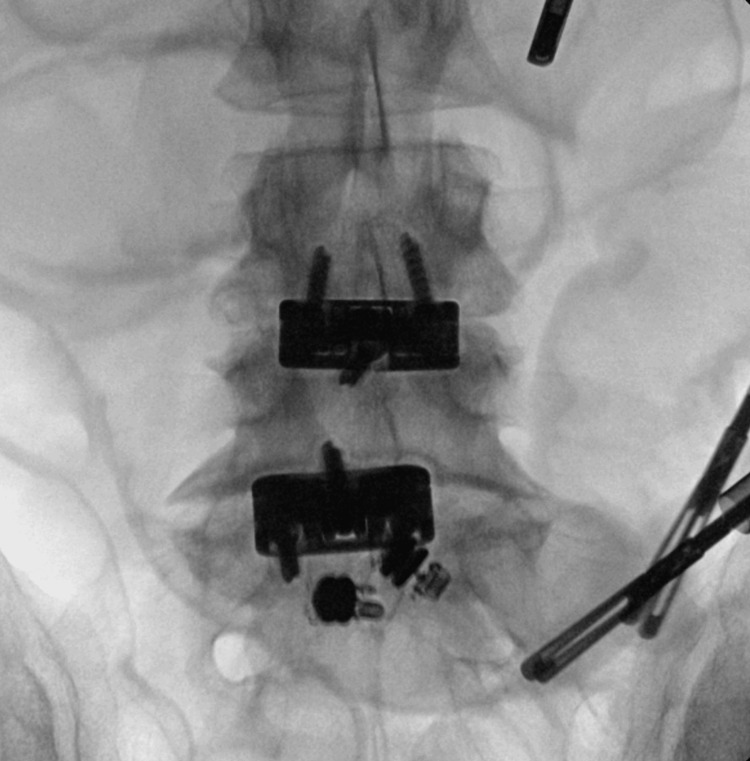
Intraoperative X-ray of video capsules before resection.

**Figure 3:**
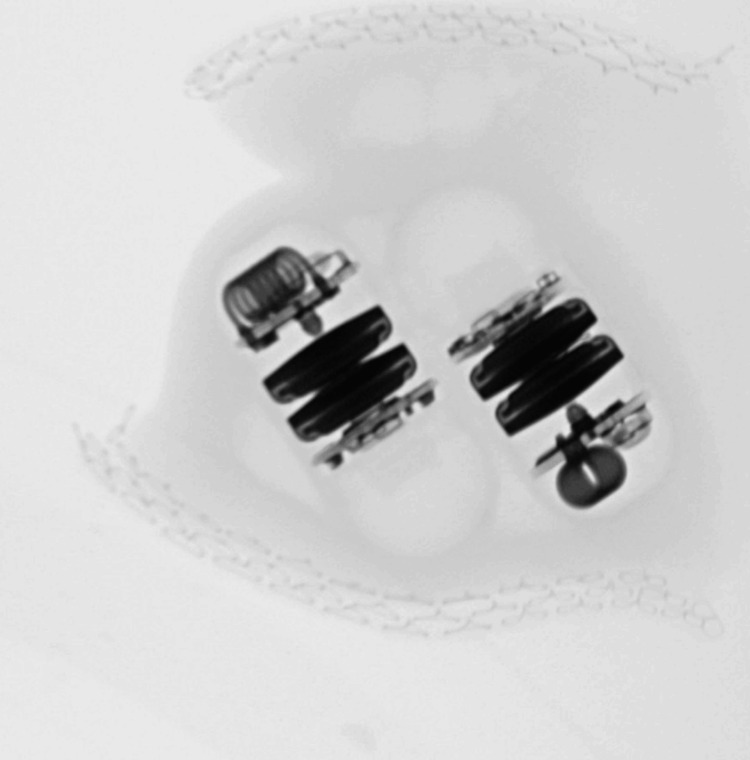
Intraoperative X-ray of video capsules post-resection.

The patient decided to proceed with surgery following an attempt at removal by lower endoscopy. Proper consent was obtained for the laparoscopic-assisted removal of small bowel foreign bodies under fluoroscopy guidance. Intraoperatively, 5 mm trocars were placed in the left upper quadrant, left mid-abdomen, and left lower abdomen. The small bowel was evaluated from the terminal ileum towards the ligament of Treitz. Fluoroscopy was used to identify the location of the capsules in the small bowel (Figure 2). The small bowel distal to it appeared strictured. Laparoscopically, the capsules were attempted to pass through this area, however they were not able to pass. At this point, it was decided to evaluate the bowel further. An incision was made infra umbilically. The small bowel was exteriorized at the location where the capsules were. Upon manual compression, the capsules did not pass through the stenosed area of the small bowel. This portion of the small bowel was then resected. Proximal and distal transection was performed with 60 mm laparoscopic echelon stapler loads. The portion of the small bowel along with the foreign bodies was transected and the mesentery was divided with the ligature device. An X-ray confirmed that foreign bodies were present in the resected bowel (Figure 3). Small bowel anastomosis was performed. The resected small bowel and foreign bodies were sent to pathology. The final histopathologic diagnosis was luminal obstruction secondary to foreign bodies (two pill cameras) with secondary submucosal edema.

Postoperative recovery was significant for fever on post-op day six. She had a 7 cm x 5.5 cm x 4 cm hepatic abscess, which was not present on the preoperative CT scan. She was admitted and treated with CT-guided percutaneous aspiration and antibiotics. She was discharged in stable condition.

## Discussion

VCE is a widely used imaging study for gastroenterology that was FDA-approved in 2001. VCE is currently indicated for obscure gastrointestinal bleeding, suspected Crohn’s disease, and small bowel tumors. The use of VCE is expanding as other indications may include abdominal pain of unclear etiology, screening for polyps in polyposis syndromes, and evaluating the small bowel for Non-Steroidal Anti-Inflammatory Drug (NSAID)--induced enteropathy. VCE is contraindicated in patients with gastroparesis, swallowing disorders, esophageal strictures, partial or intermittent bowel obstruction, and patients who are inoperable or refuse surgery [6]. Future developments in these capsule endoscopies include controlling movements, performing biopsies, and delivering drugs to targeted areas [7].

Overall, VCE is currently of low risk to patients [3]. The primary concern with this device is capsule retention. This complication is reported to occur in about 1.4% of patients of which 59% were removed surgically and 16% endoscopically [3]. According to a meta-analysis by Rezapour et al., this risk increases to about 2.2% (95% confidence interval [CI], 0.9%-5%) in abdominal pain and/or diarrhea, 3.6% (95% CI, 1.7-8.6%) in suspected IBD, and 8.2% (95% CI, 6%-11%) in established IBD. Additionally, 54% of the retentions were caused by stenosis, and 57% required surgery [4]. Unfortunately, the etiology of the stenosis was not consistently reported in the studies [4].

Other studies suggested that the rate of retention increased to 10%-20% in patients with subacute SBO and 10%-25% in patients with a small bowel tumor [8,9]. Although partial or intermittent bowel obstruction is a contraindication for VCE, some instances suggest its benefits outweigh the risks as it may lead to diagnosis and treatment of the underlying cause [10]. The use of a patency capsule is recommended for patients at increased risk because it has been shown to decrease the rate of retention [4]. 

Studies are limited in evaluating VCE for subacute bowel obstruction. The low frequency of these retentions and variability in the underlying diagnosis and history are contributing factors. Yang et al. evaluated the safety and effectiveness of VCE in 31 cases of SBO. Of these cases, no acute SBO or surgical emergencies occurred [11]. There were three cases of capsule retention; two cases had surgery for removal, and one case passed with medical therapy [11]. In cases with the potential for retention as in subacute bowel obstructions, it is important to educate patients about the possibility of surgery and ensure they are appropriate candidates if necessary. A limitation of this study is that these patients did not have any other risk factors for strictures such as NSAID-induced enteropathy or abdominal radiotherapy [11]. Additionally, a larger sample size would improve the significance.

Most retained VCEs are asymptomatic [9]. In the case of this patient, it is unclear whether her symptoms were the result of the retained capsules or the continued symptoms she had over the last five years. An initial watchful waiting period is recommended for the management of retained VCE as 35%-50% of patients will excrete the capsule without any therapy [12]. Medical therapy such as steroids and laxatives, in conditions like inflammatory bowel disease might be considered [12]. Capsule retrieval is usually recommended within 3-6 months due to the potential for perforation, obstruction, or capsule fragmentation in long-standing capsule retention [9]. 

Device-assisted enteroscopy can be used for retrieval with high safety and effectiveness [13]. A systematic review indicated a retrieval rate of 86.5% (95% CI, 75.6%-95.1%) [5]. Surgery may be the definitive management when enteroscopy fails or for treating the underlying diagnosis. For example, a few case reports demonstrated surgical intervention led to a significant diagnosis such as adenocarcinoma and Crohn’s disease [14,15]. This underscores the importance of enhancing interdisciplinary care for decisions on evaluating and treating capsule retention. In the case presented, the stenosis in the small bowel may have been clinically significant enough to have been causing partial bowel obstructions. Proper follow-up with time will determine if the removal provides long-term symptom relief. Laparoscopic-assisted surgery may be a preferred and safe method to retrieve multiple capsules and concurrently diagnose small bowel pathology [9].

## Conclusions

Capsule retention is a rare complication of VCE. However, as the utilization of VCE continues to grow, it becomes imperative for providers to know the most effective methods for capsule removal. Patients are usually asymptomatic from capsule retention. It can pass with conservative measures; however, endoscopic or surgical removal is the next step when retention persists. This case describes a patient with two retained capsules at a significant stricture in the small bowel that was successfully removed by laparoscopic-assisted surgery. VCE for obstructive symptoms may result in capsule retention, so individual risks and surgical candidacy should be considered.
